# A protocol for delivery of prehabilitation in lower limb arthroplasty in South Africa

**DOI:** 10.4102/sajp.v81i1.2037

**Published:** 2025-04-08

**Authors:** Prithi Pillay-Jayaraman, Verusia Chetty, Stacy Maddocks

**Affiliations:** 1 Discipline of Physiotherapy, School of Health Sciences, University of KwaZulu-Natal, Durban, South Africa; 2 Department of Physiotherapy, Faculty of Health Sciences, University of the Witwatersrand, Johannesburg, South Africa

**Keywords:** prehabilitation, arthroplasty, exercise, rehabilitation, hybrid

## Abstract

**Background:**

Worldwide, musculoskeletal disorders represent a global threat, and primary replacement arthroplasty is the preferred surgical treatment for late-stage arthritis. In South Africa, the waiting lists for arthroplasty are extensive and physiotherapists can have an impact on this situation by implementing prehabilitation; hence, the need to conduct research on the efficacy of such a programme.

**Objectives:**

Develop a prehabilitation programme for a resource-scarce community in South Africa.

**Method:**

Our study consisted of three phases wherein the first step entailed conducting a scoping review. The second phase was a consultation of stakeholders through semi-structured interviews and self-administered questionnaire, and the final stage was an evaluation of the effects of the prehabilitation programme by a pilot, single-blinded study on a convenient sample of patients.

**Results:**

The scoping review identified several gaps in existing programmes such as duration, mode and content of the prehabilitation programmes. Stakeholder surveys revealed a lack of knowledge and understanding of physiotherapy and prehabilitation. This highlighted the need to investigate the efficacy of a hybrid model of prehabilitation.

**Conclusion:**

Our study is novel within the South African public healthcare system, as it envisages a hybrid approach; and to construct a programme that is contextually relevant.

**Clinical implications:**

Our study aims to deliver the services in a hybrid way using telerehabilitation and face-to-face therapy which will improve access and reduce waiting times.

## Introduction

The World Health Organization (WHO) has identified osteoarthritis (OA) and rheumatoid arthritis (RA) as chronic musculoskeletal conditions that will continue to become more prevalent as the population ages (Woolf & Pfleger 2003). Data from the 2010 global Burden of Disease study provide some evidence that arthritis may be more prevalent in lower- and middle-income countries (LMICs) such as India and South Africa, than in higher-income countries. The data reflect that LMICs have 90% of the global burden of disease, but only 12% of global health spending (Brennan-Olsen et al. 2017). In South Africa, a meta-analysis done on the overall prevalence of arthritis reported that osteoarthritis was the most common form of arthritis, with a 55.1% prevalence in urban settings, and ranging between 29.5% and 82.7% in adults over 65 years of age in rural settings (Usenbo et al. 2015). There seems to be a paucity of more recent data in South Africa.

When conservative arthritis management has failed, arthroplasty is recommended as a successful treatment option for late-stage arthritis (Dunn 2012), as replacement arthroplasties have significant improvements in functional status (Räsänen et al. 2007). Total hip replacements, in particular, are referred to as the ‘operation of the century’. Within approximately 2 months after the operation, patients are able to participate in the activities of daily living that they were previously unable to do (De l’Escalopier, Anract & Biau 2016). On the other hand, waiting for an operation, using the EuroQol five-dimension (EQ-5D) questionnaire, a significant number of patients reported the experience as ‘worse than death’, according to Scott, MacDonald and Howie (2019).

The demand for arthroplasty also continues to grow. Nemes et al. (2015) revealed that it will continue to increase until a projected upper incidence level of about 469 total knee replacements per 100 000 Swedish residents, aged 40 years and older, by the year 2030. A study based in the United Kingdom, by Culliford et al. (2015) also predicted a marked increase in the need for arthroplasties. Singh et al. (2019) concluded that by 2040 the demand for Total Hip Arthroplasties (THA) will increase by 284% and the need for Total Knee Arthroplasties (TKA) will increase to 401%. Although South African-based studies estimating the future need for arthroplasty are lacking, from the global trends one may anticipate that the need will similarly increase.

The current situation in the South African public sector is that the waiting lists for arthroplasty are extensive, and the procedures are costly, with hospitals unable to cope with the demand in the public sector hospitals which serve most of the population (Abera Abaerei, Ncayiyana & Levin 2017). In the biggest academic hospitals in the region, a patient assessed as requiring an arthroplasty in 2022 could, potentially, only receive the operation in 2025, according to their waiting list registries. Similar waiting times can be found in the other academic and regional hospitals across the provinces and the country. Such a situation is not only unique to South Africa, but is prevalent in other LMICs (Dunn 2012). This long waiting period can be attributed to discrepancies between the available resources and the demand. The costs of a replacement arthroplasty are overridden by the need to prioritise trauma-related procedures (Dunn 2012).

According to the literature (Clode, Perry & Wulff 2018; Gill & McBurney 2013; McKay, Prapavessis & Doherty 2012; Saw et al. 2016; Swank et al. 2011; Topp et al. 2009; Wallis & Taylor 2011), there are various ways in which a physiotherapist can have an impact on this situation. One of the ways is to implement measures to shorten length of stay in hospital through intensive physiotherapy sessions, post-operatively. This would assist with early discharge and improve access to more beds, thereby decreasing waiting times for arthroplasty (Masaracchio et al. 2017). Another measure would be to adequately prepare, and optimise, patients prior to the surgery through physical and psycho-social measures, with a contextually relevant prehabilitation programme. Prehabilitation and pre-operative education have been shown to have a significant impact on improving outcomes, according to literature (Clode et al. 2018; Gill & McBurney 2013; McKay et al. 2012; Saw et al. 2016; Swank et al. 2011; Topp et al. 2009; Wallis & Taylor 2011).

The prehabilitation programme outlined in the literature, thus far, may not be effective in a resource-constrained public health system in the context of LMICs, because of the lack of important aspects that are pertinent to the context and profile of the patients. The gaps, identified in an overview of the literature, include limitations regarding the types of exercises and the content of education provided and the need for intensive face-to-face sessions weeks prior to the operation. In addition, available programmes do not address chronic pain problems, or the foot deformities, frequently seen in this population. Hence, there is a critical need to develop a contextually-based prehabilitation programme that will address the unique problems of the health users of a resource-constrained public health system. By conducting our study, it is hoped that positive outcomes on cost, and waiting times, may be anticipated because of the envisaged delivery of face-to-face therapy and telerehabilitation. The development of a time-, transport- and cost-effective prehabilitation programme, which is delivered in a hybrid combination of face-to-face therapy and telerehabilitation, will allow LMICs to prepare for the inevitable increase in the demand for replacement arthroplasty in the coming years.

### Aims

1. To develop a prehabilitation programme for a resource-scarce community in South Africa.
2. To investigate the effects of the programme on length of stay, physical parameters and function to optimise healthcare and rehabilitation outcomes in South Africa.

### Objectives

1. To conduct a scoping review to identify the gaps and critically inform the development of the prehabilitation programme.
2. To develop the prehabilitation and ward exercise programme through consultation with experts in orthopaedic rehabilitation and patients who are the end users.
3. To implement the prehabilitation programme and evaluate its effects on the length of stay and number of physiotherapy session needed post-operatively, physical parameters (range of motion [ROM] and muscle strength and the patient’s functional performance (walking speed, sit-stand ability, pain, activities of daily living).

## Research methods and design

The present protocol has been registered within the Open Science Framework platform (Registration ID: https://osf.io/2djau/) and our study is also awaiting registration with the National Health Research Database, South Africa. Our study is reported according to the Standard Protocol Items: Recommendations for Interventional Trials (SPIRIT) guidelines (Online Appendix 1, Addendum 1).

**FIGURE 1:**
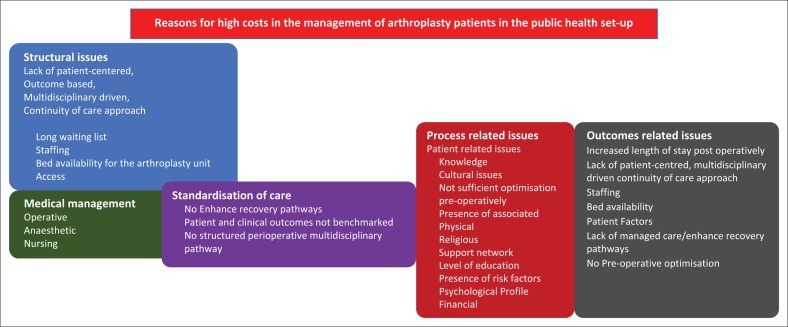
Identification and categorisation of the problem.

The main problem identified in LMICs in the management of end stage arthritis was high costs. The various factors that contribute to this problem have been identified, categorised and explained, based on the Donabedian theoretical framework (Figure 1).

Upon analysis of some of the reasons for the high cost of arthroplasty in LMICs, it was concluded that the healthcare approach is fundamentally medical. In South Africa, there is a lack of outcome-based standardisation of care and poor application of multidisciplinary, enhanced recovery pathways that have been proven to reduce the costs involved in the management of these patients in the public healthcare arena (Plenge et al. 2018). Hence, there is critical need to investigate the tenets and implementation of enhanced recovery pathways in a resource-constrained public hospital environment, which is what the current study aims to do. It is proposed that such a contextually-based prehabilitation programme needs to be developed and implemented to address structural and procedural issues in the delivery of the arthroplasty healthcare service, thereby having an impact on outcomes such as costs, inpatient length of stay and other patient-related outcomes. Problems in the structure and the process can be addressed through the implementation of a prehabilitation programme which could have an impact on patient outcomes (Plenge et al. 2019; Swenson et al. 2019).

Bates, West and Jack (2020) proposed a framework for prehabilitation services to re-engineer local perioperative services to allow for sufficient time to identify and mitigate risks by engaging prehabilitative strategies without threatening time-to-surgery. Table 1 elaborates on the steps described by Bates et al. (2020).

**TABLE 1:** Considerations in the development of a prehabilitation service.

Theoretical framework
Assess and map the surgical pathways: How might they be amended to accommodate prehabilitation? Positive change can be affected in just 2 weeks.
Gain clinical buy-in: Prehabilitation includes multiple clinical specialities and should be delivered by a specialist multidisciplinary team.
Does an existing service offer prehabilitation? There are several local health referral schemes to both gymnasia and well-being services. How effective are these? Can they align with existing rehabilitation services?
Recommendations for specific interventions are dependent on the local surgical cohort. Co-design prehabilitation services with the expert guidance of patient experience.
Quality assurance, audits, and robust data collection focussing on patient-reported outcomes are essential for service development, business case planning and quality improvement.

From the above-stated problem analysis, the methodology chosen was a prospective, mixed methods research combining qualitative and quantitative research components to facilitate heightened knowledge and allow for the legitimisation of many validities. The design employed was based on the Donabedian theoretical framework, gaining a contextual understanding through establishing broad relationships among variables uncovered, as well as a developing design paradigm wherein the results from one method will help to develop or inform the other method, and where development is broadly construed to include sampling and implementation, as well as measurement decisions (Guest 2013; Johnson 2017).

Our study comprised of three phases, and each phase had a separate methodology, target population, recruitment strategy, sampling method, size, and data analysis in order to best deliver the outcomes of the phase. The schedule of enrolment and study implementation are detailed in Table 2. Following is a description of the three phases of our study.

**TABLE 2:** Content for the schedule of enrolment, interventions and assessments.

Timepoint	Study period: 4 years		
	Phase 1	Phase 2	Phase 3
	Year 1	Year 2–3	Year 4–5
**Enrolment**	X	X	X
Eligibility screen	X	X	X
Informed consent	-	X	X
**Interventions**			
Questionnaire	-	X	X
In-depth interview	-	X	-
Prehabilitation programme	-	-	X
**Assessment**			
Hip osteoarthritis outcome score/Knee osteoarthritis outcome score	-	-	X
Timed up and go test	-	-	X
30 seconds sit stand	-	-	X
Muscle power testing	-	-	X
Goniometry	-	-	X

*Source:* Adapted from Dinu, M., Lotti, S., Pagliai, G., Napoletano, A., Asensi, M.T., Giangrandi, I. et al., 2024, ‘Effects of a chronotype-adapted diet on weight loss, cardiometabolic health, and gut microbiota: study protocol for a randomized controlled trial’, *Trials* 25(1), 152. https://doi.org/10.1186/s13063-024-07996-z

### Phase one

The first arm of our study involved conducting a scoping review to map, explore and study the breadth of information available on the topic of prehabilitation exercises and to identify gaps in the literature. The scoping review strategy, as described by Arksey and O’Malley (2005), was used and the results were published (Pillay-Jayaraman, Chetty & Maddocks 2023). The article describes in detail the various parameters to be considered in a prehabilitation programme, and an ideal version of such a programme was proposed by the author and will be the programme that will be implemented and evaluated in our study.

### Phase two

A stakeholder analysis was conducted with the patients who are the end-users of the prehabilitation programme; members of the multi-disciplinary team (doctors and nurses) who can give an opinion on the components of the programme from a multi-disciplinary team perspective; and academics in the field of orthopaedics, as well as clinicians at other similar settings who could validate the contents of the prehabilitation programme.

Consultation with patients was done through semi-structured interviews (Online Appendix 1, Addendum 2) and a self-administered questionnaire (Online Appendix 1, Addendum 3) on a sub-group cohort of patients who do not receive prehabilitation. Semi-structured interviews were used to elicit robust descriptive data on the participants’ opinions of physiotherapy, prehabilitation, their perceived challenges and suggestions for future physiotherapy. This was done to corroborate and support the quantitative information obtained from the questionnaires allowing a rich narrative between the lead researcher and the participants giving depth to the content. The guide was then developed in consultation with experts in orthopaedics and qualitative research in tertiary academic institutes in South Africa.

Psychological profiles were assessed using the Hospital Anxiety and Depression Scale (Online Appendix 1, Addendum 6). This is a valid screening tool and has been used in rehabilitation, and in this specific population (Al-Naser, James & Davies 2010; Härter et al. 2001).

A prospective single-centre study was conducted for a period of 6 months by an independent research assistant who will collect the information from the patients during their visits to the hospital’s outpatient arthroplasty clinic. All patients who were on the arthroplasty waiting list, who had been waiting for over a year for arthroplasty, and had been visiting the outpatient clinic, were canvassed to participate in this phase of our study. Every second patient in the queue who consented to participate (Online Appendix 1, Addendum 6) and indicated that they were reasonably conversant in English, were included. Those who consented to participate were taken to a quiet room and were asked to complete the questionnaires on REDCap, using the independent researcher assistant’s phone, with the research assistant being available to clarify any questions. The questionnaire (Online Appendix 1, Addendum 3) consisted of six sections in the following order: demographic information; living conditions; pain and associated problems; knowledge about the operation; knowledge about prehabilitation; and post-operative rehabilitation. The responses were categorised into yes or no answers and multiple-choice answers, and some questions had options for open-ended responses. Knowledge was scored as follows: 0 = no knowledge; 1 = answered 1–2 options (fair); 2 = answered 3–4 options (good); 3 = answered all 5 options (excellent).

The responses were collated using the Research Electronic Data Capture (REDCap) programme and then exported to Microsoft Excel and Statistical Package for the Social Sciences (SPSS). In addition, the data were sent to a statistician for analysis.

The qualitative part of our study was conducted using a convenient sample of patients awaiting arthroplasty. Data were collected from individuals who consented (Online Appendix 1, Addendum 6) using individual interviews, using the semi-structured questionnaire rubric (Online Appendix 1, Addendum 2) by a therapist experienced in conducting interviews and conversant in the patient’s language to be inclusive and to get depth in the information collected. The interviews were tape-recorded and transcribed into English using a linguist. Once the information was received from the linguist, the correctness of these verbatim transcriptions was assessed by member checking of the transcribed text, ensuring accuracy and credibility of the data (Creswell 2013; Merriam 1998). The transcribed interviews were coded and thematically analysed using the Braun and Clark (2006) method of thematic analysis. The researcher and co-authors familiarised themselves with the data by re-reading the transcripts and noting early impressions. Inductive open coding was then used to organise the data collected. Codes were developed and modified by the researcher and co-authors of our study during in-depth discussions. Codes were examined and organised into themes and were then then reviewed and defined by the researchers until consensus was achieved (Dangor 2021).

After analysis of the scoping review, questionnaires and semi-structured interviews, a second draft of the prehabilitation guide was drawn up consisting of information on four main areas: operative information, information about the type of exercises, information needed for hospital stay and post discharge information. Each chapter then elaborated on pertinent information on each of the above-stated areas. The information was presented to patients as a combination of web-based information, podcasts, pictures and videos, as well as hard copy pamphlets taking into account their preference as well.

This draft of the prehabilitation programme was then sent to arthroplasty surgeons, anaesthetists, nurses working in the arthroplasty unit, physiotherapists who worked in similar setting and academics working in the field of orthopaedics, and were considered the experts in the field of orthopaedics. The experts were invited to look at the intervention programme and commented on its appropriateness and feasibility. Thereafter, the final version of the prehabilitation programme was implemented at the phase three of our study.

#### Data analysis and data management

*Semi-structured interviews*: The handling of the data has been described in the preceding methodology section.

*Questionnaires and interviews*: Demographic information was represented by estimates of means, variance, minimum and maximum, and graphically. To compare association between variables, a statistician was contacted and statistical tests were done. Statistical analysis was done to determine if there was a relationship between variables using the Pearson’s Chi-square and/or Fisher’s exact test. The statistical test, Chi-square goodness-of-fit test, was also done to determine if any knowledge category occurs significantly more often and a *p* < 0.05 was considered statistically significant.

### Phase three

The purpose of the final arm of our study was to implement the prehabilitation programme and evaluate its effects by conducting a pilot, single-blinded study on a convenient sample of patients. All patients accessing the arthroplasty services at the orthopaedic clinics, who had been given a date to undergo primary hip or knee arthroplasty, and who will have the operation within the year, were included in our study. Those who have had previous replacements in another joint were also included with the intention to do a sub-group analysis on them. Those patients undergoing arthroplasty following trauma, or those who had juvenile arthritis, or were a revision surgery, or had participated in phase 2 of our study were not included. In addition, those patients who had had major complications post-operatively (e.g. infection) or accidents (prosthesis displacement) that required revision arthroplasty or bed rest for over 3 days were excluded.

The doctors in the arthroplasty clinic were informed about the commencement of the prehabilitation programme and notices with the above inclusion and exclusion criteria were put up so that the appropriate patients could be sent to the physiotherapy department for prehabilitation services. The prehabilitation programme was run in the physiotherapy department every Tuesday and Thursday from 12:00 to 13:00. The same gym was used for every session. Patients who consented were asked to complete the same questionnaire as for phase two (Online Appendix 1, Addendum 3). Comparing the responses of those who participate in phase two, with the responses of those who participate in the prehabilitation, will give the researcher an understanding of their experience of participation in the prehabilitation programme.

Those patients who consented to participate (Online Appendix 1, Addendum 7) in the research were asked to pick an envelope, which then randomly allocated them to the control group or the experimental group by a therapist not involved in our study, and this information was stored in a central database on the computer. Those who were in the control group underwent a battery of assessments at first contact, before the operation, and at 3-month follow-up with the doctors. The assessments are detailed next.

The consented patients were also given a general information pamphlet and a followed routine care as per the standardised protocol of the unit (Ebrahim et al. 2021). Those who are in the experimental group attended a prehabilitation class at every doctor’s visit. The prehabilitation programme was implemented as a whole, and aspects of the programme were also tested separately, and a minimum attendance of four sessions of prehabilitation was required for inclusion in our study. Patients could choose to attend their classes in person if they had sufficient doctors’ appointment sessions prior to their operation; or could attend through telehealth, for which the standard procedure is described in Ebrahim et al. (2021). All patients covered the same content irrespective of the mode of delivery. The patients were sent home with a home exercise programme and/or education pamphlet, and reminders were sent once a fortnight to ensure compliance with the programme. Patient compliance was assessed by the ability of the patient to repeat the previous class’s information. The concept of a patient-directed ward exercise programme will also be explained to the patient and the patient will be expected to do routine post operative exercises in a self-directed and independent manner. Furthermore, routine standard of care will be followed. Based on the content of the prehabilitation programme, gait and balance analysis will be done on a sub-group of patients to test the subsets of the prehabilitation programme. Independent researcher assistants conducted all the assessments and the therapist working in the orthopaedic section conducted the prehabilitation sessions, post-operative care and follow up. They were trained and oriented on the standardised assessment protocols, prehabilitation programme and standard of care, and this was recorded and monitored by using standardised notes (Online Appendix 1, Addendum 5). Three data points of assessment were used for our study: first contact and entry into the prehabilitation programme, pre-operatively and at 3 months follow-up. Each of the data points assessment included the following:

#### The Hip Injury and Osteoarthritis Outcome Score Joint Replacement (HOOS, JR) and Knee Injury Osteoarthritis Outcome Score Joint Replacement (KOOS, JR)

The HOOS and KOOS were developed as an extension of the Western Ontario and McMaster Universities’ Osteoarthritis Index (WOMAC). The tools have five separately scored sub-scales: pain; other symptoms; function in daily living (ADL); function in sport and recreation (Sport/Rec); and quality of life (QOL). The KOOS has been validated for several orthopaedic interventions such as anterior cruciate ligament reconstruction, meniscectomy and total knee replacement. The KOOS is a valid, reliable, and responsive self-administered instrument that can be used for short-term and long-term follow-up of several types of knee injury, including osteoarthritis (Roos & Lohmander 2003).

Shortened versions of the above tools are the HOOS, JR and KOOS, JR questionnaires. They measure joint-specific symptoms and focus on categories of joint pain, stiffness and function in daily living. The HOOS, JR has six questions and the KOOS JR has seven, and these tools have been validated. The research has revealed that there was high internal consistency for both HOOS, JR and KOOS, JR; and another finding was that there was near-perfect correlation with both the pain and activities of daily living/function domains of the full HOOS/KOOS and the WOMAC reporting tool (Spearman’s correlations 0.80–0.94) (Roos & Lohmander 2003). The reason for choosing the shorter version for our study was that the scoring methodology was easier to apply, compared to the HOOS/KOOS tool, and is quicker to use with patients (https://www.codetechnology.com/hoos-jr-and-koos-jr/).

#### Timed-Up and Go Test

The ‘Timed-Up and Go’ (TUG) is a modified, timed version of the ‘Get-Up and Go’ test and is a simple and inexpensive method to assess basic mobility and speed of gait, as improvement in this area is an important outcome and patient expectation. Time is measured when performing sit-to-stand and walking in the following sequence: get up from a chair (seat height 46 cm) with arm rest; walk 3 m; turn around; walk back; and sit down again. The TUG was shown to be responsive in detecting improvements after arthroplasties in the early postoperative phase (Benz et al. 2015).

#### Number of sit-to-stand in 30 s

The 30 s sit-to-stand test is also one of the recommended minimal, core, performance-based, outcome measurements in patients with osteoarthritis. Getting up from a chair is one of the activities of daily living that patients with osteoarthritis struggle with. Hence, assessing the performance of this activity post-operatively is a good indicator of outcomes of surgery and interventions. The test has been described in the literature as being sensitive enough to quantify even small changes in functional performance and is very reliable for patients with TKA (Unver et al. 2015). In this test, the patient is required to be seated on a padded 68 cm high bench, which has no arms or back. The patient then assumes an upright standing position, followed by a seated position, as many times as possible within a 30-s time interval. The number of complete stands (up from and then down to the bench) is recorded. This assessment has demonstrated high validity by being correlated with a one-repetition maximum (1RM) leg press (*r* = 0.78 men/0.71 women) and strong test–retest reliability (*r* = 0.89) (Swank et al. 2011).

#### Handheld dynamometer for muscle strength

**FIGURE 2:**
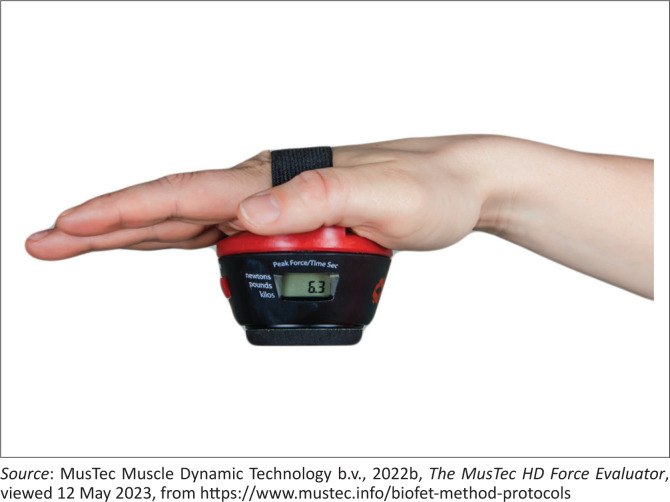
Handheld dynamometer for muscle strength.

A handheld dynamometer (Figure 2) was used to assess muscle strength in the quadriceps muscle, hip abductor muscle and the hip extensor muscle. The brand that was used was the BioFET Dynamometer which provided quantifiable measurements of a patient’s maximum muscle force, as well as the time for which that force was maintained (https://www.mustec.info/biofet-method-protocols/).

**FIGURE 3:**
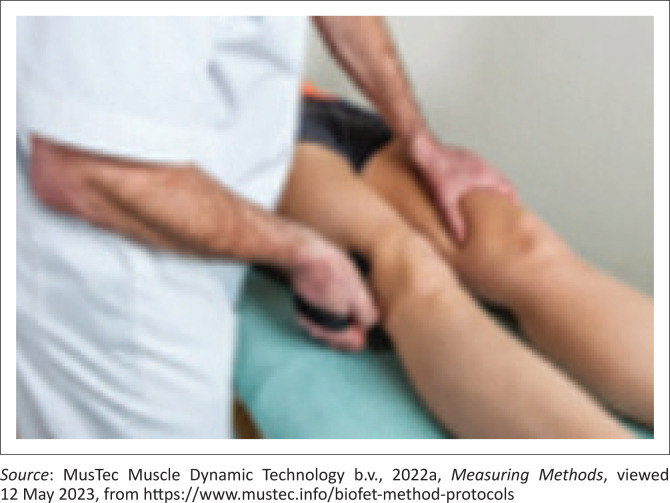
Patient position and BioFET placement for hip abduction.

A description of assessing measuring hip abduction is as follows. The patient was positioned on their back with both legs in neutral position. The therapist’s hand was placed on other leg and the BioFET was positioned on the lateral side of the leg to be assessed just above the knee (Figure 3). In order to assess muscle strength, the research assistant held the BioFET against the lateral side of the leg and exerted a force in the opposite direction of the muscle movement. The patient actively tried to hold the BioFET in place. When the physiotherapist broke through the strength of the patient, the maximum force was reached for the muscle group being tested, and the BioFET recorded the break point measurement. The isometric muscle strength value is displayed on the BioFET in the selected measurement unit.

This device has been used in clinical studies and the study conducted by Keep, Berson and Garland (2016), who reported moderate concurrent validity and good-to-high intrasession reliability across both three- and five-trial averages. Values obtained from a single trial in clinical practice also demonstrated high validity. In the study conducted by Buckinx et al. (2017), it was reported that the hand-held dynamometer demonstrated a high relative and moderate absolute reliability for all but ankle muscle groups, making the hand-held dynamometer a potential tool for research in the elderly population.

#### Range of motion: Goniometer

Range of motion was assessed using the universal goniometer, which is an instrument which measures the available ROM at a joint. It is traditionally the choice of instrument in the clinical settings because of the ease of availability and cost. Reliability of the goniometry can be ensured by careful measurement technique and by using the same tester (Ekstrand et al. 1982).

In recent years, researchers and clinicians have measured joint range by downloading an application (app) on a smart phone. A study done by Milanese et al. (2014) reported that the universal goniometer, when compared to a smartphone goniometer, was reliable; and the agreement was high between these instruments. A similar finding was reported by Jones et al. (2014), who found there were no significant differences in the measurements of individual knee joint angles between a smart app goniometer and universal goniometer. Based on the preceding information, a universal goniometer will be used for the purposes of our study, and active and passive ROM will be assessed following the guidelines set out by Norkin and White (2016) for the affected limb; and the un-affected limb will be cleared using quick tests.

#### Data analysis and data management of the prehab programme

All the raw data were collected as hard copies, and stored in a sealed box. This information was then transferred into Microsoft Excel and SPSS. Descriptive information was represented by estimates of means, variance, minimum and maximum, and also graphically using box plots and graphs. To test the hypothesis and ascertain if the prehab programme was effective, Spearman’ correlation coefficient was used to assess correlations between two quantitative data. Values of *P* smaller than 0.05 were considered statistically significant.

## Discussion

The significance and value of this current work is that our study is novel within the South African public healthcare system. With the proposed advent of National Health Insurance, our study will assist in adding to the body of knowledge and establish a standard of practice in arthroplasty. Based on the literature, this is the only study in South Africa to engage with the stakeholders and experts prior to structuring a prehabilitation programme. This will allow for a knowledge base that is contextually relevant. Most studies in the literature consider the prehabilitation programme 2 months prior to the operation, and this puts the patient in a very unrealistic situation. Our study envisages studying the effects of the programme on different subsets of participants in the prehabilitation programme following a natural order of events, and our study is practical and is independent of staffing changes.

This is the only study incorporating a standardised educational approach in the public health context, and the only study and hospital in South Africa to deliver services through hybrid approach of face-to-face therapy and telerehabilitation. Despite telerehabilitation growing as a mode of healthcare delivery in recent years in developed countries, its use in public health settings in LMICs has been limited because of a lack of awareness and reservations (Odole et al. 2015). Our study will, therefore, enable us to explore the implementation and application of telerehabilitation and offer the option of a hybrid model of care in a public health setting to provide continuity of care, by providing alternative options that will enhance compliance.

The outcomes of our study will allow us to explore a hybrid model of care and its effectiveness in improving access to care; improved quality of care, and short- and long-term outcomes; patient and therapist satisfaction; and the best use of human resources. The results from our study will, therefore, explore both clinical outcomes as well as perceptions of prehabilitation and telerehabilitation to gain an in-depth understanding of its efficacy, thereby determining its role in a public health setting (Ebrahim et al. 2021).

There are some limitations in our study, where the methodology follows mixed methods research design and is not a randomised controlled study and, in addition, double blinding is not possible. The sampling method in phase three of our study uses a convenient sampling and it is recommended that a randomised controlled study be done in subsequent studies with a larger sample and consider the health structure as a whole, that is, including private health setup to allow for a standardised implementation. The programme should also be investigated and implemented in the main regional South African languages to get more in-depth analysis and further enhancing access.
